# Astrocytic and microglial cell functions in neuroinflammatory diseases and their animal models

**DOI:** 10.3389/fncel.2025.1708775

**Published:** 2025-12-08

**Authors:** Kei Hashimoto, Mari Gotoh, Hiroko Ikeshima-Kataoka

**Affiliations:** 1 Institute for Human Life Science, Ochanomizu University, Bunkyo, Japan; 2 Faculty of Medical Technology, Teikyo University, Itabashi, Japan; 3 Kitasato University School of Frontier Engineering, Kanagawa, Japan

**Keywords:** Alzheimer’s disease, astrocyte, lipid droplet, microglia, mouse, Parkinson’s disease, TBI, zebrafish

## Abstract

Neurodegenerative diseases are frequently accompanied by inflammatory responses and alterations in lipid metabolism, both of which are believed to negatively affect neural regeneration in mammals. In addition to immune cells, glial cells such as astrocytes and microglia contribute significantly to these inflammatory processes, and it is now recognized that lipid droplet accumulation and cholesterol metabolism are dysregulated in these glial cells. Consequently, recent studies have examined inflammation and lipid metabolism from the standpoint of glial cell function; however, effective therapeutic strategies remain unestablished. By contrast, in zebrafish, a teleost species, robust neural regeneration occurs within a short period after injury to the telencephalon or spinal cord. In this review, we aimed to identify candidate functional factors by comparing mouse and zebrafish disease models and to explore molecules with potential therapeutic relevance for mammalian neurological disorders.

## 1 Introduction

In mammals, nerve regeneration after injury is generally limited. Therefore, brain and spinal cord injuries, along with many neurodegenerative diseases, remain incurable, and the replacement of lost neurons is extremely challenging. The activation of glial cells such as microglia and astrocytes, triggered by neuronal degeneration, is believed to induce inflammation and subsequently lead to the formation of glial scars, which inhibit neuronal regeneration (Gao et al., 2013; Lukacova et al., 2021). Moreover, recent studies have shown that lipid droplet accumulation and dysregulated cholesterol metabolism in glial cells further contribute to neuronal stress and hinder regeneration. By contrast, other studies suggest that astrocytes and microglia perform essential functions in forming glial scars, which act as protective barriers that limit the spread of inflammation following central nervous system (CNS) injury (Sofroniew, 2015; Sims and Yew, 2017; Adams and Gallo, 2018). Lipid droplet formation in astrocytes is likewise thought to play a protective role for neurons.

Furthermore, activated microglia have been reported to phagocytose dead neurons, thereby clearing cellular debris and creating space for potential tissue remodeling (Jurga et al., 2020).

Our previous studies have demonstrated that astrocytic and microglial activation is crucial for recovery from blood–brain barrier disruption after brain injury (Hashimoto et al., 2018; Ikeshima-Kataoka and Yasui, 2016) and have also revealed the neuroprotective roles of tenascin-C, one of the extracellular matrix glycoprotein, secreted by astrocytes in the mouse brain (Ikeshima-Kataoka et al., 2008; Nakashima et al., 2021). More recently, the neuroprotective effects of vitronectin, one of the extracellular matrix protein, have also been reported (Yamashita et al., 2025). Based on these findings, astrocytes and microglia may represent promising targets for promoting neuronal regeneration in mammals.

In contrast to mammals, zebrafish exhibit robust regenerative ability in the brain, including the telencephalon and spinal cord, without forming glial scars after injury (Marques et al., 2019; Diotel et al., 2020; Kolb et al., 2023). However, microglial activation and inflammation are indispensable for regeneration following telencephalic injury in zebrafish (Kyritsis et al., 2012; Palsamy et al., 2023).

In this review, we compare recent findings from mouse and zebrafish neurodegenerative disease models and focus on astrocytes and microglia to identify candidate factors that may provide clues for promoting neural regeneration in mammals.

## 2 Clinical features, genetics, and neuropathology of Alzheimer’s disease

The clinical manifestations of Alzheimer’s Disease (AD) are classified into early-onset and late-onset forms, distinguished primarily by the age at symptom onset. One widely accepted diagnostic standard is the guideline for AD diagnosis developed by the National Institute on Aging and the Alzheimer’s Association (McKhann et al., 2011).

Although familial AD has been documented, more than 90% of cases represent sporadic AD, with familial cases typically manifesting earlier in life. Two major genetic mutations associated with familial AD have provided key insights into disease mechanisms. The first involves dominant mutations in *APP*, which encodes the amyloid precursor protein (Aβ precursor peptide) (Li et al., 2019). The second involves missense mutations in *PSEN1* and *PSEN2*, which encode subunits of the *γ*-secretase complex responsible for cleaving APP into Aβ (Sun et al., 2017). In addition, *APOE* ε4 has been identified as the strongest genetic risk allele for AD. Large-scale twin studies indicate that genetic factors influence the timing of AD onset, although non-genetic risk factors also play important roles (Gatz et al., 2006). Approximately 62% of people with sporadic AD carry the *APOE* ε4 allele (Rebeck et al., 1993). Furthermore, polygenic risk score analyses incorporating *APOE* have demonstrated that carriers of *APOE* ε4 develop AD approximately 4–5.5 years earlier than non-carriers (de Rojas et al., 2021). Less common genetic risk alleles include *ABCA7*, *ABI3*, *BIN1*, *CR1*, *TREM2*, and *SORL1* (de Rojas et al., 2021). Recent mouse models of AD are summarized in Table 1.

**Table 1:** Alzheimer’s disease models of mouse.

Categories	Methods	Target cells	Results	Target molecules	References
APP/PS1	Double-transgenic mouse expressing chimeric mouse/human APP with the Swedish mutation (K670N/M671L) and mutant human PSEN1 lacking exon 9.	Microglia	Axl and Mertk regulate microglial engulfment of Aβ plaques and synapses.	Axl, Mertk (TAM receptors)	Huang et al. (2021)
					Huang and Lemke (2022)
		Microglia	The TREM2–APOE pathway mediates microglial accumulation around senile plaques to phagocytose Aβ.	APOE, TREM2	Krasemann et al. (2017)
					Parhizkar et al. (2019)
		Astrocyte	Astrocytes take up Aβ for detoxification into urea but produce the neurotoxic byproduct GABA.	ARG1, ODC1	Ju et al. (2022)
		Astrocyte	Upregulated Nrf2 expression in astrocytes attenuates Aβ deposition.	Nrf2	Jiwaji et al. (2022)
PS2APP	Transgenic mouse expressing mutant human PSEN2 (N141I) and APP with the Swedish mutation (K670N/M671L).	Astrocyte	Astrocytic STIM1 reduction induces declines in Ca^2^⁺ signaling and synaptic plasticity.	STIM1	Lia et al. (2023)
PS2APP TauPS2APP	PS2APP: Transgenic mouse expressing mutant human PSEN2 (N141I) and APP with Swedish (K670N/M671L) TauPS2APP: Transgenic mouse expressing mutant human Tau (P301L), PSEN2 (N141I) and APP with Swedish (K670N/M671L)	Astrocyte	Reactive astrocytes express high levels of C4b.	C4b	Lee et al. (2021)
TauPS2APP	Transgenic mouse expressing mutant human Tau (P301L), PSEN2 (N141I), and APP with the Swedish mutation (K670N/M671L).	Astrocyte Microglia	TREM2 regulates microglial and astrocytic phagocytosis of synapses.	TREM2	Dejanovic et al. (2022)
P301S-Tau/ PS19	Transgenic mouse expressing the P301S mutant form of human tau.	Microglia	TREM2 also regulates microglial reactivation.	TREM2	Gratuze et al. (2020)
		Microglia	Tau⁺ neurons exposing phosphatidylserine (PS) trigger microglial phagocytosis.	MFGE8	Brelstaff et al. (2018)
		Astrocyte Microglia	Astrocytes and microglia mediate C1q-dependent synapse elimination.	C1q	Dejanovic et al. (2022)
5 × FAD	Transgenic mouse expressing APP with the Swedish (K670N/M671L), Florida (I716V), and London (V717I) mutations, along with PSEN1 carrying the M146L and L286V mutations.	Microglia	TREM2 regulates the activation of microglia into the DAM phenotype.	TREM2	Keren-Shaul et al. (2017) and Zhou et al. (2020)
		Astrocyte	Astrocyte-secreted IL-3 promotes microgliosis and facilitates Aβ plaque clearance.	IL-3	McAlpine et al. (2021)
5 × FAD CRND8	5 × FAD: Transgenic mouse expressing APP with Swedish (K670N/M671L), Florida (I716V), and London (V717I) mutations, and PSEN1 with M146L and L286V mutations CRND8: Double-transgenic mouse expressing APP with both the Swedish (KM670/671NL) and Indiana (V717F) mutations.	Microglia	Microglial processes make stable contact with and encapsulate Aβ plaques.	ATP-binding cassette subfamily A member 1(ABCA1)	Condello et al. (2015)

The neuropathological hallmarks of AD include severe atrophy of the medial temporal lobes, particularly the hippocampus and entorhinal cortex, and the accumulation of extracellular senile plaques composed of Aβ, together with intracellular neurofibrillary tangles composed of phosphorylated tau (Whitwell, 2010; Tcw and Goate, 2017). Deposition levels of these aggregates are positively correlated, with Aβ deposition generally preceding tau pathology (Cras et al., 1991).

### 2.1 The neuroimmune system and microglia in AD patients and mouse models

A prominent feature of the AD brain is microglial activation, which has been actively investigated in recent years due to advances in transcriptomic technologies. Integration of AD genome-wide association studies (GWAS) with myeloid-specific epigenomic and transcriptomic datasets has shown that AD risk alleles are enriched in active enhancers of microglia (Novikova et al., 2021), suggesting that regulation of microglial function contributes to AD pathogenesis.

Microglial reactivation is essential for the phagocytosis of senile plaques, neurofibrillary tangles, and apoptotic neurons, thereby helping to maintain brain homeostasis in AD. The mechanisms underlying microglial dynamics have been characterized using various mouse models. In five familial Alzheimer’s disease (5 × FAD) and Center for Research in Neurodegenerative Diseases 8 (CRND8) mice, microglial processes have been shown to stably contact and envelop Aβ plaques. Interestingly, plaques covered by microglia tend to be more compact and exhibit lower Aβ42 affinity, leading to reduced dystrophic neurite formation (Condello et al., 2015). These findings indicate that microglia act as protective barriers against neurotoxicity.

Several mechanisms mediating microglial Aβ phagocytosis have been described. TAM receptors such as Axl and Mertk on microglia interact with Gas6 ligands surrounding Aβ plaques to promote phagocytosis in APP/PS1 mice (Huang et al., 2021). Consistent with this, Mertk deficiency in APP/PS1 mice results in hippocampal hyperexcitability due to impaired clearance of apoptotic neurons and defective synaptic pruning, which in turn leads to recurrent seizure activity and premature death (Huang and Lemke, 2022). Thus, microglial phagocytosis is crucial for eliminating damaged neurons and protecting brain function.

Tau pathology also induces microglial phagocytosis. In P301S-Tau mice, neurons containing tau inclusions expose phosphatidylserine, which serves as an “eat-me” signal to activate microglial phagocytosis and facilitate the removal of damaged neurons (Brelstaff et al., 2018). Moreover, tau secreted by neurons promotes microglial proliferation, which accelerates the clearance of damaged neurons in primary rat neuron–glia cultures (Pampuscenko et al., 2020). Collectively, these findings strongly suggest that microglia mitigate tau toxicity by removing compromised neurons.

Several lines of evidence indicate that microglial reactivation in AD is progressively regulated by TREM2. Large-scale exome sequencing of individuals with AD has identified rare damaging variants in *TREM2*, which encodes a receptor critical for microglial reactivation (Holstege et al., 2022). *TREM2* governs the transition from homeostatic microglia to disease-associated microglia (DAM), a subset proposed to represent the reactivated state in neurodegenerative diseases. Specifically, the DAM subset was identified by single-cell RNA sequencing of 5 × FAD mouse brains and is characterized by elevated expression of *Trem2*, *Cx3cr1*, *Apoe*, *Cd9*, and *Cst7*, among other genes. DAM clusters around Aβ plaques and displays high phagocytic activity. Notably, microglial activation occurs through a two-step process: an initial TREM2-independent phase that initiates DAM activation, followed by a *TREM2*-dependent phase in which microglia acquire robust phagocytic activity (Keren-Shaul et al., 2017). Supporting the regulatory role of TREM2, loss-of-function mutations in TREM2 markedly impair microglia-mediated elimination of excitatory and inhibitory synapses, ultimately leading to attenuation of tau pathology and neurodegeneration (Gratuze et al., 2020; Dejanovic et al., 2022).

Several TREM2 variants, including R47H and R62H, alter the binding capacity of TREM2 to its ligands and are associated with an increased risk of AD (Song et al., 2017). APOE has been identified as a ligand for TREM2, and this interaction enhances the microglial phagocytic clearance of apoptotic neurons (Rebeck et al., 1993; Bailey et al., 2015). Consistent with these findings, the TREM2-APOE signaling pathway has been recognized as a major regulator of microglial phenotypic changes in AD. Phagocytic microglia demonstrate the most prominent upregulation of *Apoe* expression in APP/PS1 microglia. Furthermore, individuals with AD carrying TREM2 R47H and R62H variants exhibit reduced microglial clustering around Aβ plaques, which consequently promotes greater Aβ plaque deposition. In contrast, individuals with AD and TREM2 haplodeficiency display similar levels of microglial reactivation irrespective of APOE ε4 allele status (Krasemann et al., 2017), suggesting that APOE functions downstream of TREM2. Additionally, Trem2 deficiency-induced microglial quiescence in APP/PS1 models reduces ApoE localization to Aβ plaques, indicating that microglia serve as a pivotal source of plaque-associated ApoE (Parhizkar et al., 2019).

Given the preponderance of evidence supporting microglial reactivation as a gatekeeper of the neuroimmune system, we believe that a timely review of these studies will provide deeper insights into the physiological functions of microglia. However, the role of microglia in AD remains controversial. Single-cell RNA sequencing of human AD microglia has revealed a spectrum of subtypes: some subtypes show downregulation of AD pathology-related genes, others show upregulation of Aβ-related genes, and still others exhibit alterations in genes associated with neurofibrillary tangles (Olah et al., 2020). This transcriptional diversity suggests that individual AD microglia possess distinct molecular programs that contribute to barrier functions. It is also important to emphasize that there are marked species-specific differences in AD microglial biology. For example, comparison of *Trem2^−/−^*;5 × FAD mice with individuals carrying *TREM2* mutations demonstrated that human AD exhibits upregulated expression in only part of the DAM signature, despite TREM2 being required for microglial reactivation (Zhou et al., 2020). Furthermore, RNA sequencing of microglia isolated from the human AD cortex revealed that most AD risk genes, termed human AD microglia (HAM) genes, which show microglia-specific preferential expression, overlap substantially with those of aged human microglia. Interestingly, the expression of DAM genes identified in the 5 × FAD mouse strain remains unchanged in human AD. By contrast, differentially expressed gene sets identified in aged microglia of PVM and PS2SPP mice are significantly enriched in HAM (Srinivasan et al., 2020). Therefore, it is necessary to interpret mouse and human microglial profiles separately. Moreover, even within mouse models, the functional properties of microglia may vary depending on the specific AD strain used.

### 2.2 Astrocyte function in AD patients and mouse models

Reactive astrocytes, referred to as disease-associated astrocytes (DAAs) by analogy to DAMs, also contribute to neuroinflammation in AD (Habib et al., 2020). However, their mechanisms of action are diverse and remain incompletely understood. Although RNA sequencing of fluorescence-activated cell sorting (FACS)–purified astrocytes derived from human AD samples shows no significant changes in the expression of genes associated with AD risk (Srinivasan et al., 2020), Single-cell RNA sequencing of multiple human brain regions—including the entorhinal cortex, inferior temporal gyrus, dorsolateral prefrontal cortex, secondary visual cortex, and primary visual cortex—spanning normal aging to severe AD progression shows that astrocytes can be classified into several subsets, with transcriptomic changes in synapse-related and stress response–related genes occurring along vulnerable neural networks and AD pathology stages, respectively (Serrano-Pozo et al., 2024). Astrocyte reactivation may be regulated by complex dynamics involving both spatial and temporal axes during the pathogenesis of AD.

Astrocytes play key roles in regulating synaptic plasticity in AD. They exhibit excitability through dynamic changes in intracellular Ca^2+^ concentration, acting as regulators of the brain environment. In the PS2APP mouse model of AD, Ca^2+^ signaling in astrocytes is markedly reduced due to the downregulation of the Ca^2+^ sensor protein STIM1, leading to astrocyte-dependent long-term synaptic plasticity decline and subsequent memory loss. Notably, STIM1 overexpression in astrocytes completely restores astrocytic Ca^2+^ signaling and synaptic plasticity in PS2APP mice (Lia et al., 2023).

Collectively, astrocytic activity contributes to memory decline through dysregulation of synaptic plasticity in AD. Consistent with this, astrocytes in TauP301S mice have been reported to engulf excitatory and inhibitory synapses in a C1q-dependent manner, compensating for microglial impairment of TREM2-related phagocytosis (Dejanovic et al., 2022). Similarly, in individuals with AD, astrocytes engulf synapses through recognition of MFG-E8 via αvβ5 integrin (Tzioras et al., 2023). Although it remains unclear how astrocytes coordinate synaptic connectivity and engulfment through Ca^2+^ signaling, astrocyte reactivity clearly contributes to memory decline in AD. Microglial dynamics are considered essential for astrocyte reactivation. Recent high-resolution spatial transcriptomics of APP^NL-G-F^ mice revealed that astrocytic responses to Aβ plaques are heterogeneous. A neurotoxic astrocytic phenotype emerges in a microglial density–dependent manner, characterized by increased GABAergic signaling and decreased glutamatergic signaling, suggesting that reactive astrocytes contribute to the disruption of the excitatory–inhibitory balance in neuronal circuits (Mallach et al., 2024). Overall, these findings suggest that astrocytes display neurotoxic properties toward neuronal circuits while interacting with microglia and neurons in AD. Moreover, overexpression of tau in astrocytes has been reported to reduce neurogenesis and impair neuronal circuits, indicating that tau accumulation in astrocytes alone may be sufficient to induce AD-like symptoms (Richetin et al., 2020). Further elucidation of the mechanisms underlying astrocyte-related neuronal circuit coordination is warranted.

Here, to gain deeper insight into astrocyte function in AD, findings on astrocytic Aβ clearance are introduced. Astrocyte-released IL-3 has been reported to stimulate microglia to assemble around Aβ and tau aggregates and clear them through microglial phagocytosis in the 5 × FAD mouse brain (McAlpine et al., 2021). Moreover, exposure of Aβ to human astrocytes derived from stem cells of healthy donors promotes microglial Aβ phagocytosis, whereas astrocytes carrying the APOE4 allele and treated with Aβ show reduced microglial Aβ uptake, suggesting that astrocytes enhance microglial Aβ clearance in an APOE4-dependent manner (Lee et al., 2025). In addition to regulating microglial dynamics, astrocytes modulate Aβ accumulation through Nrf2 expression. Translating ribosome affinity purification sequencing of astrocytes from APP/PS1 and MAPT^P301S^ mice revealed enriched expression of Nrf2, which encodes a stress-activated cytoprotective protein, in both models. Astrocytic Nrf2 expression reduces Aβ accumulation and tau phosphorylation (Jiwaji et al., 2022). These findings suggest that astrocytes act as neuroprotective regulators of Aβ and tau aggregation. Conversely, other evidence indicates that astrocytes can promote tau aggregation. It is well known that tau tangle accumulation is initiated by Aβ aggregation in AD. Interestingly, some Aβ-positive patients with AD do not develop tau pathology. Positron emission tomography analyses revealed that only patients classified as reactive astrocyte–positive, based on plasma GFAP detection, show tau tangle accumulation, suggesting that astrocyte reactivity is an important upstream regulator of early tau pathology induced by Aβ (Bellaver et al., 2023). Thus, astrocytes in AD regulate Aβ and tau proteinopathy in complex and multifaceted ways.

Astrocytes themselves secrete neurotoxic factors. Astrocytes play essential roles in maintaining various metabolic pathways, including glucose, lipid, and amino acid metabolism (Zhang et al., 2023), One report indicated that reactive astrocytes in both patients with AD and APP/PS1 mice display dual beneficial and toxic roles within the urea cycle. Reactive astrocytes uptake Aβ to detoxify it into urea; however, an excessive byproduct of this cycle, *γ*-aminobutyric acid (GABA), exerts toxic effects that impair memory (Ju et al., 2022). In addition, PS2APP and TauPS2APP mice show an increased number of reactive astrocytes expressing high levels of the complement factor C4b, a key mediator of synaptic degeneration (Lee et al., 2021; Zheng et al., 2025). Together, these findings indicate that astrocytes are sources of neurotoxic factors that accelerate AD progression.

## 3 Clinical features, neuropathology, and genetics of Parkinson’s disease

The clinical features of Parkinson’s Disease (PD) include motor symptoms such as tremor, rigidity, and bradykinesia, and diagnosis is made according to the criteria established by the International Parkinson and Movement Disorder Society (Postuma et al., 2015).

A defining neuropathological characteristic of PD is the presence of Lewy bodies, which are intracytoplasmic inclusions composed of full-length *α*-synuclein (*α*-Syn) in dot- or thread-like forms. Lewy bodies are primarily observed in the neuronal cytoplasm of the substantia nigra in individuals with PD (Spillantini et al., 1997, 1998).

Although familial PD has been documented, more than 90% of cases are idiopathic. Large-scale GWAS in PD have identified strong associations with *SNCA*, which encodes *α*-Syn, and *MAPT*, which encodes tau. In addition, *LRRK2*, *PARK16*, *GBA*, and *TMEM175* have been reported as genetic risk alleles for PD (Simón-Sánchez et al., 2009; Nalls et al., 2014). These genes have been implicated as potential mediators of PD pathogenesis through proteogenomic network analysis (Doostparast Torshizi et al., 2024). Furthermore, higher expression of ApoE has been reported in melanized neurons of the substantia nigra (Wilhelmus et al., 2011). The major mouse models of PD are summarized in Table 2.

**Table 2:** Parkinson’s disease models of mouse.

Categories	Methods	Target cells	Results	Target molecules	References
*α*-synuclein preformed fibril injection	Transgenic mouse expressing mutant human PSEN2 (N141I) and APP with the Swedish mutation (K670N/M671L).	Microglia	Depletion or inhibition of microglial activation attenuates α-synuclein (α-Syn) accumulation and dopaminergic neuron loss in the substantia nigra.	CSF1R	Thi Lai et al. (2024)
		Astrocyte	α-Syn preformed fibrils induced C4 secretion from neurons, which leads to astrocytic inflammation.	C4	Zou et al. (2025)
*DJ-1^−/−^* (*PARK7^−/−^*)		Microglia	DJ-1 inhibits the NF-κB pathway to maintain microglial quiescence.	p65, NF-κB	Lin et al. (2021)
I2-2 G2-3 H5 O2	Transgenic mice expressing human wild-type α-synuclein (I2-2), A53T mutant α-synuclein (G2-3 and H5), and A30P mutant α-synuclein (O2).	Astrocyte	A53T mutant α-Syn disrupts astrocytic Ca^2^⁺ signaling.		Nanclares et al. (2023)
*LRRK2^−/−^* LRRK2 G2019S-knock in		Astrocyte	Elevated extracellular clusterin impairs α-Syn clearance in astrocytes.	clusterin	Filippini et al. (2021)
					Filippini et al. (2025)
LRRK2 G2019S-knock in		Microglia	The LRRK2 G2019S mutation suppresses the NF-κB pathway through PKA dysregulation, promoting cytokine production in microglia.	PKA	Russo et al. (2018)
LV-FLEX-*SNCA^G420A^*	Bilateral injection of LV-FLEX-SNCAG420A into CX3CR1-CreERT2 mice.	Microglia	Microglia-specific α-Syn overexpression promotes dopaminergic neuron loss through oxidative molecule production.	Oxidative molecule	Bido et al. (2021)
MPTP	Systemic administration of MPTP hydrochloride combined with probenecid.	Astrocyte	LCN2 interferes with α-Syn binding to the 24p3R receptor, impairing α-Syn uptake in astrocytes.	LCN2, 24p3R	Jiao et al. (2025)

### 3.1 The neuroimmune system and microglia in PD patients and mouse models

Single-cell RNA sequencing of the substantia nigra from individuals with PD has revealed a significantly higher proportion of microglia (Martirosyan et al., 2024), suggesting that microglial function is altered in PD. A previous study reported that microglial depletion attenuates *α*-Syn accumulation and dopaminergic neuronal loss in the substantia nigra of PD model mice (Thi Lai et al., 2024). These findings support the idea that microglia exert neurotoxic effects more prominently in PD.

The role of microglia in PD is gradually being elucidated. Studies have shown that *α*-Syn–deficient microglia display higher cytokine production and reduced phagocytic activity, suggesting that *α*-Syn regulates microglial inflammatory responses (Austin et al., 2006). The uptake of α-Syn by microglia is regulated by leucine-rich repeat kinase 2 (LRRK2). Microglia carrying the LRRK2-G2019S mutation exhibit reduced motility and enhanced cytokine production due to inhibition of focal adhesion kinase activity and activation of the nuclear factor (NF)-κB pathway, respectively (Choi et al., 2015; Russo et al., 2018). On the other hand, α-Syn can also enhance the neurotoxic properties of microglia. Microglia-specific overexpression of α-Syn promotes their reactivation, leading to dopaminergic neuronal loss through increased production of reactive oxygen species (Bido et al., 2021). In addition, *α*-Syn interacts with microglia via the receptor for advanced glycation end products to promote cytokine secretion (Long et al., 2022). Collectively, these findings demonstrate that *α*-Syn can drive microglial neurotoxic activation.

In addition, it is possible that *α*-Syn contributes to neuronal cell death, but this effect can be mitigated through microglial interaction via tunneling nanotube formation. Specifically, microglia receive *α*-Syn from neurons through F-actin–dependent tunneling nanotubes and, in turn, donate intact mitochondria to neurons to reduce oxidative stress *in vitro* (Scheiblich et al., 2024). Interestingly, microglia containing *α*-Syn can transfer it and exchange mitochondria with neighboring microglia through F-actin–dependent intercellular connections, thereby escaping cell death induced by oxidative toxicity (Scheiblich et al., 2021). These findings suggest that microglia support the homeostasis of both neurons and themselves by inhibiting *α*-Syn exposure and supplying mitochondria. Notably, the above microglia–neuron communication system via tunneling nanotubes has been confirmed *in vitro*; therefore, further demonstration *in vivo* is anticipated.

Through a mechanism distinct from *α*-synuclein toxicity, DJ-1 also modulates microglial inflammation in PD. Knockdown of DJ-1 has been reported to enhance dopamine-induced cytokine production, oxidative stress, and phagocytosis in microglia via p65 nuclear translocation of the NFκB pathway, ultimately contributing to dopaminergic neuronal loss (Trudler et al., 2014; Lin et al., 2021). In contrast, under LPS-induced inflammatory conditions, DJ-1 deficiency suppresses microglial activation (Lind-Holm Mogensen et al., 2024), suggesting that DJ-1 represents a potential risk factor for PD.

### 3.2 Astrocytic function in PD patients and mouse models

Single-cell transcriptomic analysis of the substantia nigra from individuals with PD has also revealed a higher proportion of astrocytes (Martirosyan et al., 2024), suggesting that astrocytic as well as microglial functions play key roles in PD.

The crucial role of astrocytes in PD pathology is the clearance of *α*-synuclein (*α*-Syn). Interestingly, primary astrocytes from healthy rodent ventral midbrain and cortex have been shown to inhibit neuronal *α*-Syn aggregation. Specifically, administration of ventral-midbrain- or cortex-derived astrocytes into the midbrain of mice injected with α-Syn adeno-associated virus (AAV) and preformed fibrils to generate a PD model reduces α-Syn aggregation and neurodegeneration in the substantia nigra (Yang et al., 2022). Moreover, treatment with *α*-Syn preformed fibrils promotes *α*-Syn uptake in primary astrocyte cultures (Filippini et al., 2021). Therefore, healthy astrocytes exert therapeutic effects on PD progression. Conversely, PD astrocytes exhibit reduced *α*-Syn clearance ability, the molecular mechanisms of which are being elucidated. One mechanism of astrocytic *α*-Syn clearance involves regulation by extracellular chaperones. LRRK2 is known to regulate *α*-Syn uptake by astrocytes as well as microglia. LRRK2 G2019S knock-in mice show enhanced expression of the extracellular chaperone clusterin through the activity of microRNA miR-22-5p, leading to impaired *α*-Syn clearance in astrocytes. Notably, transfection of miR-22-5p improves astrocytic *α*-Syn clearance by reducing clusterin production (Filippini et al., 2021, 2025). Another mechanism of astrocytic α-Syn clearance involves the interaction between *α*-Syn and its membrane receptors. Injection of 1-methyl-4-phenyl-1,2,3,6-tetrahydropyridine (MPTP) or α-Syn preformed fibrils into mice enhances expression of lipocalin-2 (LCN2) in astrocytes of the substantia nigra. LCN2 inhibits α-Syn binding to its membrane receptor 24p3R, thereby impairing astrocytic *α*-Syn uptake (Jiao et al., 2025). Collectively, astrocytic dynamics determine PD pathogenesis and progression through the regulation of α-Syn accumulation. It is possible that *α*-synuclein (α-Syn) is closely associated with astrocyte dynamics, although many aspects remain unknown. This possibility is supported by several studies reporting that expression of *α*-Syn with the A53T mutation in mice alters Ca^2+^ signaling in astrocytes, which may contribute to changes in both astrocytic and neuronal functions (Nanclares et al., 2023). Moreover, although *α*-Syn aggregation is well known to occur in neurons, astrocytes that internalize *α*-Syn can also accumulate it. During PD progression, astrocytic *α*-Syn first appears mainly in the amygdala at early stages, followed by the cingulate and entorhinal cortices, and finally affects the cerebellum and substantia nigra at advanced stages (Otero-Jimenez et al., 2025). Furthermore, astrocytes have been reported to acquire distinct forms of *α*-Syn truncated at the N-terminus between residues 21–33 and the C-terminus between residues 100–102, and phosphorylated at tyrosine 39 (Altay et al., 2022). Interestingly, *α*-Syn–laden astrocytes secrete abundant extracellular vesicles (EVs) as a result of lysosomal dysfunction *in vitro*. Consistent with this, plasma from PD patients contains more astrocyte-derived GLT-1^+^ EVs enriched in *α*-Syn (Wang et al., 2023), suggesting that astrocyte-derived EV content in plasma may serve as a potential biomarker for clinical diagnosis of PD.

Several lines of evidence demonstrate that PD astrocytes display a proinflammatory state. Single-cell RNA sequencing of the substantia nigra from PD patients indicates that CD44^+^ astrocytes are expanded, and CD44 promotes neuroinflammatory signaling through the JAK/STAT pathway in astrocytes (Ma et al., 2025), suggesting that astrocytes acquire neurotoxic properties in PD. It is thought that the neuroinflammatory state of astrocytes is induced by extracellular *α*-Syn. *α*-Syn fibrils are known to activate cytokine production in primary human astrocytes through receptor interacting protein kinase 1 (RIPK1)- and receptor interacting protein kinase 3 (RIPK3)-dependent NF-κB signaling, thereby promoting neuronal cell death (Chou et al., 2021). In addition, injection of α-Syn preformed fibrils into mice induces C4 secretion from neurons, leading to astrocyte-mediated, inflammation-related neuronal death (Zou et al., 2025). Furthermore, treatment with oligomeric *α*-Syn induces an inflammatory state in astrocytes derived from human stem cells, characterized by enhanced secretion of inflammatory factors, including IFNγ, IL-1β, and TNFα. Notably, IFNγ promotes expression of the A-to-I RNA-editing mediator ADAR1 p150 isoform, thereby sustaining the inflammatory state in astrocytes (D’Sa et al., 2025). Together, astrocyte-related inflammation contributes to neurodegeneration in PD.

Moreover, stem cells derived from PD patients demonstrate that enhanced TLR2 activity induces neurotoxic factor secretion in parallel with autophagy-dysfunction-related *α*-Syn aggregation in astrocytes (Weiss et al., 2024), suggesting that dysregulation of lysosomal degradation may promote neurotoxic factor release in PD astrocytes. Notably, a recent study reported that mice injected with *α*-Syn–AAV into the substantia nigra exhibit a proinflammatory astrocytic state in the midbrain but not in the striatum (Basurco et al., 2023). suggesting that astrocytic inflammation is activated in a brain-region-dependent manner.

It is also possible that PD astrocytes affect blood–brain barrier (BBB) dynamics as well as neuronal function. The substantia nigra in PD patients shows morphological BBB alterations, particularly a reduction in vessel area. RNA-sequencing data indicate that astrocytes derived from iPS cells carrying the PD-related LRRK2 G2019S mutation exhibit transcriptomic changes in angiogenesis-related and proinflammatory genes. Consistent with this, BBB coculture and histological analyses demonstrate that astrocytes with the LRRK2 G2019S mutation promote abnormal increases in BBB permeability, caused by morphological changes in brain microvascular endothelial cells (de Rus Jacquet et al., 2023). Insights into BBB dynamics in PD remain limited; therefore, further research is required.

## 4 Clinical features of traumatic brain injury

The severity of traumatic brain injury (TBI) is quantified using several evaluations, with the Glasgow Coma Scale being the most widely applied. This scale is scored based on eye, motor, and verbal responses (Teasdale and Jennett, 1974). TBI severity is influenced by the impact of both primary and secondary brain injuries. Primary brain injury refers to the immediate physical damage, including axonal loss and hemorrhage. Secondary brain injury, in contrast, arises from edema, ischemia, and inflammation-related gliosis and neuronal cell death, which develop minutes to days after the primary insult. Secondary brain injury is widely considered to exert a major impact on TBI outcomes. Consistent with this concept, individuals with severe TBI, including contusions, exhibit more pronounced gliosis and aggravated neuronal damage compared with those with mild TBI (Feichtenbiner et al., 2025). Furthermore, the degree of secondary injury depends on the severity of the primary injury. For example, macrophage interaction with fibrin, which accumulates near sites of extravasated blood components, activates inflammatory responses in TBI (Dean et al., 2024). Taken together, these findings highlight a strong correlation between the impact of primary brain injury and the extent of secondary brain injury. To mimic the characteristics of TBI, various murine models have been developed. Major murine models of TBI are summarized in Table 3.

**Table 3:** Traumatic brain injury models of mouse.

Categories	Methods	Target cells	Results	Target molecules	References
Controlled cortical impact	Open-head brain injury induced using a mechanical impactor to deliver a precisely controlled object strike to the exposed cortex.	Microglia	Deletion of CD36 polarizes microglia toward a neuroprotective phenotype.	CD36, Traf5, MAPK	Hou et al. (2024)
		Microglia	The Fas–FasL pathway promotes microglial polarization into a neurotoxic phenotype.	Fas, FasL	Xia et al. (2024)
		Microglia	DAM-like microglia are required for neurological recovery.	TREM2	Wang L. et al. (2025)
		Microglia	Sterol synthesis in microglia provides essential lipids to oligodendrocytes, promoting remyelination.	LXR	Li et al. (2024)
		Microglia	Brain injury induces C3 deposition in the dorsolateral geniculate nucleus, leading to microglia-mediated synaptic loss.	C3	Borucki et al. (2024)
		Astrocyte	Astrocyte-derived extracellular vesicles deliver miRNA-382-5p to neurons, causing mitochondrial dysfunction and neuronal apoptosis.	miRNA-382-5p	Hu et al. (2024)
		Astrocyte	PD-L1⁺ reactive astrocytes accumulate at the lesion site and prevent excessive neuroimmune activation.	PD-1, PD-L1	Gao et al. (2022)
		Astrocyte	Astrocyte-derived exosomal long non-coding RNA suppresses microglial activation.	long non-coding RNA, SMAD7	He et al. (2023)
		Astrocyte	IL-1 signaling exerts protective effects by regulating astrogliosis.	IL-1R1	Vincent et al. (2023)
		Astrocyte	Astrocytes expressing AQP4 accelerate glial scar formation.	AQP4	Zou et al. (2025)
Fluid percussion	Open-head brain injury generated by applying a brief fluid pulse to the intact dura through a craniotomy.	Microglia	Microglial secretion of IFN drives chronic inflammation.	STING, IFN	Packer et al. (2024)
					Packer et al. (2025)
Stab injury	Open-head brain injury created using a needle to produce localized cortical damage.	Astrocyte	Drebrin expression in astrocytes supports glial scar formation.	Debrin	Schiweck et al. (2021)
Weight drop	Closed-head, diffuse brain injury induced by dropping a calibrated weight onto the skull to replicate concussive impact.	Astrocyte	TLR4-expressing microglia interact with astrocytes to promote synaptic loss, while astrocyte-secreted thrombospondin-1 promotes synaptic recovery.	TLR4, TSP1	Rosa et al. (2021)

### 4.1 The neuroimmune system with microglia in TBI patients and mouse models

Relieving secondary brain injury is crucial for improving the prognosis of TBI. Importantly, microglia act as critical regulators of secondary injury, demonstrating both neurotoxic and neuroprotective functions. Supporting this concept, microglial depletion has been reported to suppress proinflammatory factor production and neurodegeneration after brain injury (Henry et al., 2020; Packer et al., 2024), suggesting that the neurotoxic role of microglia is particularly influential in TBI.

Consistent with the importance of microglial activation in TBI, single-cell transcriptomic studies have identified a transformation of microglial phenotypes toward a proinflammatory state after injury. Single-cell RNA sequencing of human brain tissue from individuals with severe TBI revealed a higher proportion of microglia, representing the cell type with the most substantial population change (Garza et al., 2023). Notably, in mouse models, a distinct proinflammatory microglial cluster was expanded within 24 h of injury and persisted for up to 6 months (Jha et al., 2024), indicating that microglial activation occurs rapidly after trauma and sustains chronic inflammation. Together, these findings support the hypothesis that proinflammatory microglial activation contributes to the worsening of TBI prognosis.

**Figure 1:**
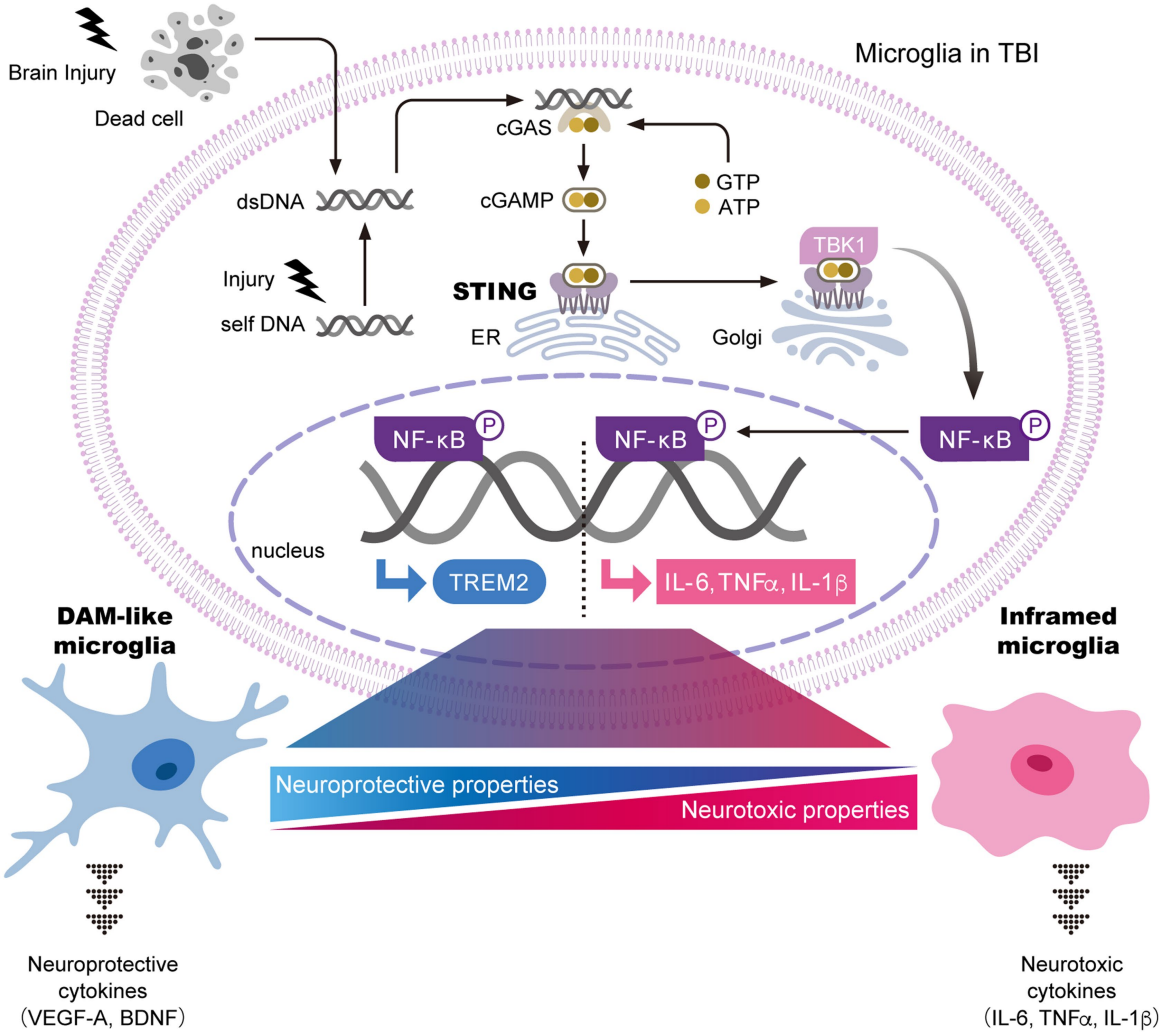
The STING pathway is a key regulator of microglial transformation into both neurotoxic and neuroprotective phenotypes. In general, the STING pathway is essential for the innate immune response and inflammatory signaling. In this pathway, damaged cell-derived exogenous or self-DNA activates cytoplasmic cGAS. The binding of cGAS to DNA triggers the production of cGAMP, which interacts with STING in the ER and activates it. Activated STING translocates to the Golgi apparatus, where it promotes the phosphorylation of NF-κB through TBK1. NF-κB then translocates into the nucleus to induce transcription of neurotoxic cytokines, including IL-6, TNFα, and IL-1β, thereby driving the transformation of microglia into an inflammatory phenotype with neurotoxic properties. In parallel, nuclear NF-κB promotes the transcription of TREM2, which induces the transformation of microglia into DAM-like microglia. DAM-like microglia secrete neuroprotective cytokines that support neuronal survival. Taken together, brain injury is thought to regulate the balance between neuroprotective and neurotoxic microglial properties through the STING pathway. Further identification of key factors that determine microglial states after brain injury is expected. The figure is a modified version of Hu et al. (2022). ATP; adenosine triphosphate, BDNF; brain-derived neurotrophic factor, cGAMP; cyclic GMP-AMP, cGAS; cyclic GMP-AMP synthase, DAM; disease-associated microglia, dsDNA; double stranded DNA, ER, endoplasmic reticulum; GTP; guanosine triphosphate, IL; interleukin, NF-κB; nuclear factor-κB, STING; stimulator of interferon genes, TBK1; tank-binding kinase 1, TREM2; triggering receptor expressed on myeloid cells 2, VEGFA; vascular endothelial growth factor A.

Pertinent to the mechanism of microglial activation in TBI, several studies have reported that the stimulator of interferon genes (STING) pathway acts as a critical regulator of the transformation of microglia into a proinflammatory state (Figure 1). Activation of STING leads to the production of inflammatory factors (Abe and Barber, 2014; Yum et al., 2021). Consistent with these findings, deficiency of STING has been shown to inhibit microglial activation and morphological changes after brain injury (Packer et al., 2024). These data suggest that the STING pathway contributes to chronic inflammation in TBI. In addition, microglia-specific STING deficiency attenuates both microgliosis and proinflammatory factor expression (Packer et al., 2025). Taken together, the STING pathway plays a critical role in driving inflammatory responses through the sensing of damaged cell-derived DNA in microglia after TBI. Notably, although the STING pathway upregulates the transcription of interferon (IFN)- and NF-κB-mediated genes (Abe and Barber, 2014; Yum et al., 2021), deficiency of IFNAR1, the receptor for IFN, does not affect microglial activation after brain injury (Packer et al., 2025). Therefore, it is possible that STING pathway-induced chronic inflammation is mediated primarily through NF-κB-dependent production of interleukin (IL)-6, tumor necrosis factor (TNF)*α*, and IL-1β, rather than IFN signaling. Further studies are required to elucidate the detailed mechanisms of microglial activation after brain injury.

It is recognized that a subset of activated microglia demonstrate neuroprotective functions, not only neurotoxic properties, after brain injury. One such neuroprotective subset is referred to as DAM-like cells, which show marked upregulation of DAM markers such as *Cd9*, *Cst7*, *Lpl*, *Csf*, and *Timp2*. Interestingly, the severe neurodegeneration observed in *Trem2^−/−^* mice appears to result from the absence of DAM-like cells with neuroprotective properties, including the production of repair-related cytokines and the phagocytosis of cellular debris (Wang L. et al., 2025). In other words, DAM-like cells function to attenuate neurodegeneration in TBI. However, the boundary and transition between neuroprotective and neurotoxic microglia are difficult to define. The STING pathway has been shown to induce upregulation of TREM2 expression in addition to proinflammatory factor expression (Dabla et al., 2022), suggesting that STING may regulate transformation into both neurotoxic and neuroprotective microglial phenotypes (Figure 1). In contrast, STING deficiency in TBI models results in only a neuroprotective phenotype (Packer et al., 2024, 2025). This finding suggests either that TREM2-related DAM transformation does not depend on STING signaling or that neurotoxic microglia emerge before neuroprotective populations after brain injury. Therefore, it is possible that functional variations exist within the roles of activated microglia in TBI, which are now being more comprehensively characterized through transcriptomic and proteomic analyses.

Aside from inflamed and disease-associated microglia (DAM-like), the classifications of neurotoxic M1 and neuroprotective M2 phenotypes are the most well characterized. A marked increase in microglial numbers is observed within 7 days after brain injury, consisting of a mixture of M1 and M2 phenotypes. After this period, the population shifts toward a predominance of M2 microglia (Morganti et al., 2016). The mechanisms underlying M1-M2 polarization are becoming increasingly clear. Polarization toward the M1 type is mediated by the Fas–FasL pathway after brain injury (Xia et al., 2024). In addition, CD36, which is one of the molecules robustly upregulated after brain injury, promotes microglial polarization toward the M1 phenotype through the Traf5-MAPK pathway (Hou et al., 2024). Taken together, microglial polarization towards M1 is regulated by multiple pathways. It is important to note that the concept of a transitional microglial activation system between neurotoxic and neuroprotective states has become more widely accepted than the traditional M1–M2 polarization model, as comprehensive analyses have revealed the diversity of microglial phenotypes in TBI.

Activated microglia strongly influence neuronal dynamics in TBI. In individuals with severe TBI, a significant increase in microglia apposing neuronal somata has been observed, suggesting that brain injury enhances microglia–neuron communication (Feichtenbiner et al., 2025). A well-established form of microglia–neuron communication is mediated by microglia-derived proinflammatory cytokines, which promote neurodegeneration. M1 microglia secrete proinflammatory mediators such as IL-1 and TNF, thereby amplifying the neurotoxic environment (Xia et al., 2024; Packer et al., 2025). In addition to cytokine signaling, microglial phagocytic activity contributes to neurodegeneration through synaptic loss following brain injury. It has been demonstrated that brain injury induces neuronal C3 deposition around synapses, which triggers microglial phagocytosis of synaptic structures and consequently reduces neuronal activity (Borucki et al., 2024). These microglial properties collectively exacerbate neurodegeneration. By contrast, DAM-like cells exhibit neuroprotective functions. Specifically, DAM-like cells have been reported to secrete anti-inflammatory mediators that suppress neurodegeneration in controlled cortical impact models (Wang L. et al., 2025). Furthermore, sterol synthesis is activated by the liver X receptor (LXR) pathway in DAM-like cells, supplying essential lipids to oligodendrocytes to promote remyelination after brain injury (Li et al., 2024). Considering their diverse roles, including the production of pro- and anti-inflammatory mediators, phagocytosis of debris and synapses, and provision of essential trophic factors, microglia play a central role in regulating neuronal dynamics both directly and indirectly in TBI.

### 4.2 Astrocytic function in TBI patients and mouse models

Single-cell RNA sequencing of human TBI brain tissue indicates that astrocytes undergo transcriptomic alterations after brain injury, although their responses are less pronounced than those of microglia (Garza et al., 2023; Jha et al., 2024). Moreover, single-cell RNA sequencing of mouse brains with closed-head injury shows that the astrocyte population decreases immediately after injury, followed by expansion around the lesion over 6 months (Garza et al., 2023). Another study reports that cortical injury influences hippocampal neural stem cell dynamics, which regulate neurogenesis and astrogliogenesis. Controlled cortical impact promotes proliferation of neurons, astrocytes, and neural stem cells in the hippocampus, while single-cell RNA sequencing demonstrates that brain injury enhances neurogenesis derived from neural stem cells at the expense of astrogliogenesis (Bielefeld et al., 2024). In brief, astrocyte expansion after TBI is not derived from hippocampal neural stem cells. The expanded astrocyte population is thought to perform diverse functions, and the details of these functions in TBI are gradually being elucidated.

One critical function of astrocytes in TBI is glial scar formation, which limits damage caused by physical trauma and subsequent inflammation. It has been reported that stab wounds induce immediate upregulation of the actin-binding protein Drebrin in astrocytes, supporting glial scar formation through modulation of Rab8^+^ membrane tubule trafficking (Schiweck et al., 2021). However, glial scars have dual roles in TBI repair, as astrocytes expressing the water channel aquaporin-4 (AQP4) accelerate scar formation after TBI through cytotoxic swelling, consequently increasing post-traumatic seizure susceptibility (Lu et al., 2021). Collectively, astrocyte-associated glial scars can both minimize tissue damage and disturb neural activity in TBI.

Astrocytes have also been recognized as mediators of neuroinflammation through cytokine-dependent glia–neuron communication (Vincent et al., 2023). Although microglia exhibit more extensive transcriptomic alterations after brain injury than astrocytes, several genes—such as those related to interferon (IFN) signaling and major histocompatibility complex class I antigen presentation—show transcriptional changes in both cell types after injury (Todd et al., 2021). Therefore, astrocytes also undergo inflammatory state–related cellular changes after brain injury. While astrocytes are known to produce neurotoxic factors (Todd et al., 2021), they also possess anti-inflammatory properties. For example, astrocyte-derived exosomal noncoding RNA 4933431K23Rik suppresses injury-induced microglial activation and excessive microglial SMAD7 expression, thereby inhibiting NF-κB signaling and alleviating neuroinflammation (He et al., 2023). Furthermore, astrocytes modulate the neuroimmune response through the programmed cell death protein-1 (PD-1)/programmed cell death ligand-1 (PD-L1) immune checkpoint pathway. PD-L1^+^ reactive astrocytes accumulate around lesions and prevent excessive neuroimmune responses by suppressing infiltration of PD-1^+^ immune cells (Gao et al., 2022). Collectively, astrocytes propagate diverse molecular signals that influence both repair and injury processes after TBI, although the underlying mechanisms remain to be fully elucidated.

Astrocytes also affect neural functions in TBI. For instance, toll-like receptor 4 (TLR4)–expressing microglia interact with astrocytes to promote synaptic loss after brain injury, whereas astrocyte-derived thrombospondin-1 supports synaptic recovery, suggesting that reactive astrocytes contribute to synaptic remodeling in TBI (Rosa et al., 2021). Additionally, microRNAs (miRNAs) have emerged as another mediator of glia–neuron communication. Astrocyte-derived EVs deliver miR-382-5p to neurons, leading to mitochondrial dysfunction and neuronal apoptosis (Hu et al., 2024). Considering their diverse roles—including glial scar formation, pro- and anti-inflammatory signaling, synaptic remodeling, and regulation of neuronal survival—astrocytes play a crucial role in orchestrating brain repair after TBI.

## 5 Astrocyte-mediated lipid droplet accumulation and cholesterol metabolism in neuroinflammatory diseases

Advances in neuroscience technologies in the 2000s revealed that LDs, the storage organelles for energy-rich neutral lipids, are present in glial cells of the brain. Moreover, increased LD formation and accumulation have been associated with aging and several neuropathologies, including AD, PD, and multiple sclerosis (Ralhan et al., 2021; Smolič et al., 2022) and TBI (Li et al., 2023). Notably, Alois Alzheimer’s 1907 translated report already described one characteristic finding in the brain of a patient with AD as the presence of “adipose saccule” in glial cells (Stelzmann et al., 1995). Subsequent studies demonstrated that LD formation in astrocytes protects neurons from various cellular stresses, including oxidative, metabolic, and hypoxic stress (Sultana et al., 2013; Liu et al., 2017; Smolič et al., 2021), while also limiting lipid accumulation in neurons. However, when LD accumulation becomes excessive, astrocytes undergo phenotypic changes and become neurotoxic lipid-accumulating reactive astrocytes (LARAs) (Chen et al., 2023). At the same time, microglia exhibit reduced phagocytic function, increased production of reactive oxygen species, and enhanced secretion of inflammatory cytokines (Marschallinger et al., 2020). Oleic acid and palmitic acid induce LD accumulation in primary cultured astrocytes (Nakajima et al., 2019; Kwon et al., 2017). and these astrocytes subsequently secrete inflammatory cytokines that promote microglial migration and activation (Kwon et al., 2017). Furthermore, recent work has shown that microglia, which contribute to Aβ clearance in the early stages of AD, develop defects in Aβ phagocytosis when LDs accumulate, thereby accelerating amyloid pathology and leading to neuronal damage (Prakash et al., 2025). Thus, while LD formation in glial cells initially exerts protective functions for neurons, excessive LD accumulation in glial cells contributes to pathological processes in the brain. Importantly, LD accumulation precedes Aβ deposition. Because LD dysregulation is implicated in multiple brain diseases, investigating the mechanisms of LD accumulation and degradation is critical for understanding the underlying causes of these disorders.

**Figure 2:**
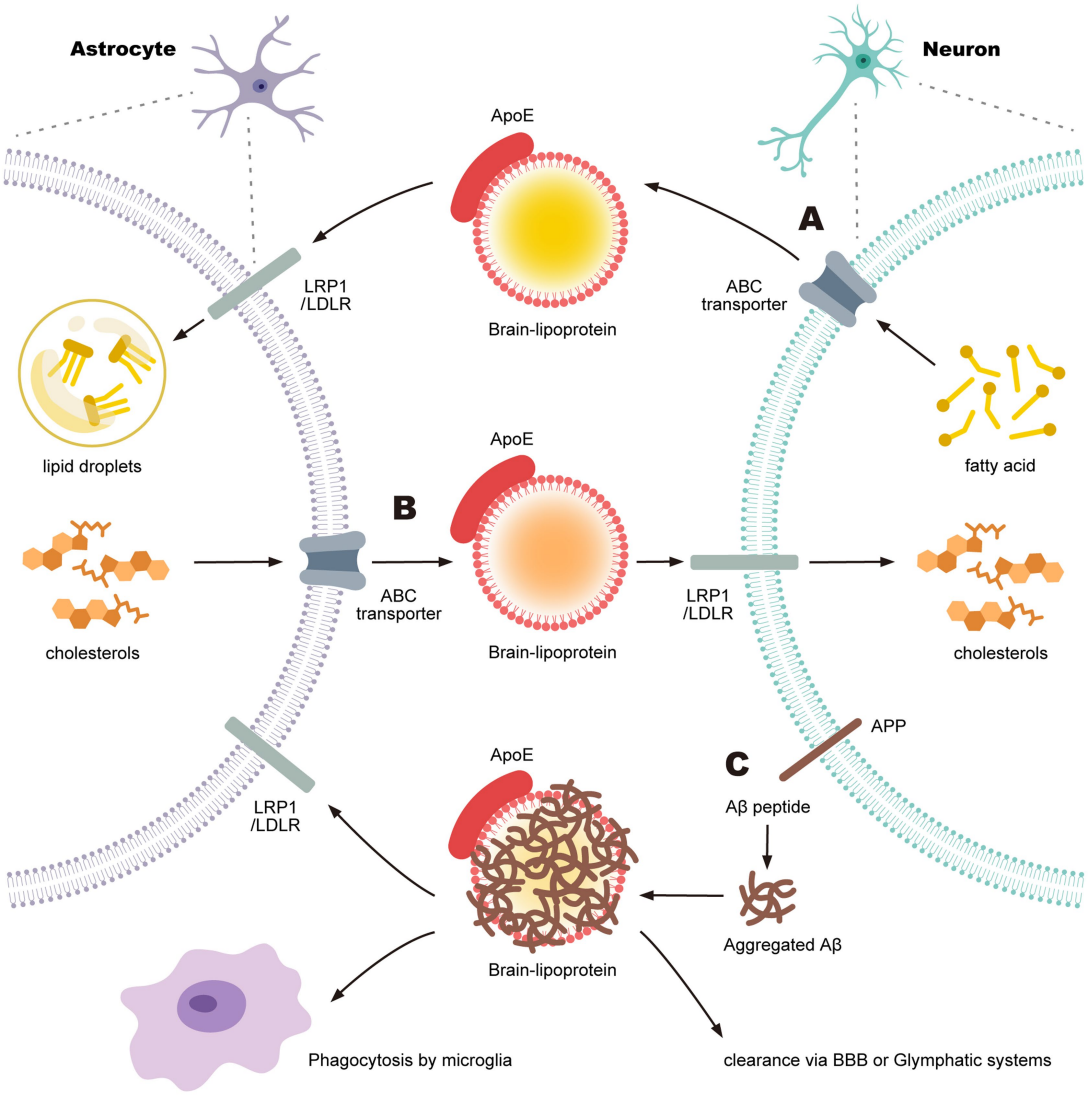
ApoE-regulated lipid transport between neurons and astrocytes and Aβ clearance. **(A)** Triacylglycerol (TAG) synthesized in neurons is transferred to astrocytes via ApoE-containing brain lipoproteins. **(B)** Cholesterol synthesized in astrocytes is transferred to neurons via ApoE-containing brain lipoproteins. **(C)** Aggregated Aβ binds to ApoE-containing brain lipoproteins, facilitating its clearance.

Then, here we focus on the transport of triacylglycerols (TAGs) and cholesterol, the major components of LDs, between neurons and astrocytes (Figure 2). LDs contain TAGs and cholesterol esters in their core, surrounded by a phospholipid monolayer that incorporates free cholesterol and various proteins.

TAGs consist of a glycerol backbone esterified with three fatty acids (FAs). In astrocytes, FAs for TAG synthesis are supplied by neurons via ApoE-associated lipid particles (Ioannou et al., 2019) and from the bloodstream through the BBB. Neurons express ApoE during oxidative stress, and it has been suggested that ApoE facilitates lipid transfer (Ralhan et al., 2021) (Figure 2A). Additionally, astrocytes can *de novo* synthesize FAs from lactate via acetyl-CoA. FAs and diacylglycerol are then esterified to form TAGs by the enzyme diacylglycerol acyltransferase (DGAT), which is localized to the endoplasmic reticulum (Lan et al., 2023). The resulting TAGs are packaged into LDs in the astrocyte cytoplasm. These processes are especially active when neurons are under cellular stress.

Cholesterol exists in two principal forms: free cholesterol and cholesterol ester (CE). In the bloodstream, most cholesterol is esterified, with approximately two-thirds present as CE and one-third as free cholesterol. In contrast, in the brain, only about 1% of cholesterol is esterified (Hyuk Yoon et al., 2022), highlighting a fundamental difference in cholesterol biology between the CNS and peripheral tissues (Stubbs and Smith, 1984). Cholesterol in phospholipid bilayers influences both the fluidity and rigidity of lipid membranes. In the brain, many cells contain extensive phospholipid bilayer regions, including neurons with their long axons, astrocytes and microglia with their numerous processes, and oligodendrocytes with their myelin sheaths. Proper membrane fluidity and rigidity are essential for synaptic transmission mediated by vesicle fusion and budding, as well as for the activity of several transmembrane proteins, including receptors and ion channels. The brain requires 20–25% of the body’s total cholesterol, making it the tissue with the highest cholesterol concentration. However, because the BBB is impermeable to cholesterol, brain cholesterol levels are largely independent of peripheral cholesterol. Instead, most cholesterol in the brain is synthesized *de novo* by astrocytes (Shin et al., 2024). Once synthesized, cholesterol is secreted from astrocytes through ATP-binding cassette (ABC) transporters, esterified to CE by lecithin-cholesterol acyltransferase (LCAT), and transferred to neurons via ApoE-containing high-density lipoprotein (HDL)-like particles (i.e., brain lipoproteins) (Tsujita et al., 2024). These CE-containing ApoE particles are then taken up by neurons through LRP1 and LDLR (Jin et al., 2019) (Figure 2B). The cholesterol obtained by neurons supports neurogenesis and synaptogenesis, whereas excess cholesterol is converted into 24-hydroxycholesterol (24-OH), which can cross the BBB and be excreted. Thus, ABC transporters in astrocytes, LRP1/LDLR in neurons, and ApoE synthesized by astrocytes are all essential for intercellular cholesterol transport between astrocytes and neurons. Dysfunction of these proteins is linked to impaired lipid metabolism and LD accumulation in both neurons and astrocytes (Liu et al., 2010; Windham et al., 2024; Wang S. et al., 2025). Lipoproteins serve as vehicles to transport insoluble molecules, such as CE. Several neurodegenerative diseases, including AD and PD, are characterized by the accumulation of protein aggregates and the formation of insoluble plaques in the brain, ultimately leading to neuronal death. Aggregated A*β* is one of the key pathological factors in AD. Although insoluble, aggregated Aβ binds to ApoE-containing brain lipoproteins, and the resulting Aβ-ApoE complexes are eliminated from the CNS into systemic circulation via the BBB or the glymphatic system (Figure 2C) (Campos-Peña et al., 2022). Therefore, ApoE plays a critical role in Aβ clearance and is considered a promising therapeutic target for AD and other neurodegenerative disorders.

Recent advances in mass spectrometry have enabled detailed characterization of fatty acid chain composition. For example, studies have revealed that unsaturated FAs are decreased in the AD brain (Yin, 2023). As lipidomic research progresses and the components of brain lipids are identified with greater precision, our understanding of lipid contributions to neurodegeneration will become more comprehensive. Interestingly, it has also been reported that the composition of brain lipids in zebrafish is affected by environmental temperature (Maffioli et al., 2024). Moreover, studies using zebrafish have demonstrated that elimination of accumulated LDs is necessary to promote recovery during regeneration following TBI (Zambusi et al., 2022). Therefore, LD clearance facilitates neuroregeneration, a key finding from zebrafish studies that may provide translational insights for mammalian models. In the following chapters, we focus on zebrafish models of several neurodegenerative diseases.

## 6 Zebrafish as a model for glial cell research

Zebrafish, a teleost fish, offers several advantages compared to other animal models. They have a short life cycle, reaching sexual maturity within 3 months, and females can lay hundreds of eggs at a time. Furthermore, external development and the transparency of embryos and larvae allow for easy manipulation, and cellular processes can be imaged *in vivo* using fluorescent reporters (Dooley and Zon, 2000).

Comparative genomic analyses have shown that 76% of genes implicated in human diseases through GWAS are conserved in zebrafish (Howe et al., 2013). In addition, the zebrafish brain shares structural and functional similarities with the mammalian brain, including the presence of the same major cell types: neurons, astrocytes, oligodendrocytes, and microglia (Schmidt et al., 2013). A detailed review on the functional roles of immune cells and their responses in the zebrafish CNS has previously been published (Oosterhof et al., 2015).

As an introduction to this section, we first describe the glial cells and their primary functions in zebrafish, which are the focus of this review.

### 6.1 BBB in zebrafish

At the BBB, zebrafish BBB form tight junctions composed of ZO-1 and claudin-5 (CLDN5), similar to mammals, and maintain close contact with pericytes (O’Brown et al., 2018). In zebrafish, two paralogs, a CLDN-5a and -5b, are expressed in cerebrovascular endothelial cells; however, Li et al. demonstrated that CLDN-5a functions as the ortholog of mammalian CLDN5 (Li et al., 2022). Taken together, CLDN5 is highly conserved from fish to humans, making it an important factor in the BBB.

Key molecular mechanisms of BBB formation have also been elucidated using zebrafish. Notch3 signaling has been shown to be essential for pericyte proliferation, regulation of pericyte numbers, and BBB integrity in zebrafish (Wang et al., 2014). Furthermore, Hübner et al. employed high-resolution *in vivo* imaging and demonstrated that Wnt signaling is required for brain angiogenesis and BBB formation (Hübner et al., 2018). Inhibition of Wnt signaling with IWR-1 produced severe vascular defects and hemorrhages in the zebrafish larvae. In addition, America et al. revealed that GPR124/Reck function implicated in Wnt ligand-specific cellular responses in developing brain vasculature in both zebrafish and mice, providing evidence for functional conservation of these pathways across vertebrates (America et al., 2022). By contrast, double-transgenic zebrafish expressing glut1β:mcherry and plvap: EGFP clarified that VEGF signaling is critical for CNS angiogenesis but not required for Wnt/β-catenin-dependent BBB properties (Fetsko et al., 2023). They further demonstrated that activation of Wnt/β-catenin signaling occurs independently of VEGF in brain endothelial cells. Thus, Wnt signaling in zebrafish BBB formation remains controversial.

Neuronal contributions to BBB development have also been identified. Neurons secrete Spock1, which induces BBB integrity. Knockout of SPOCK1 in zebrafish and mice resulted in BBB leakage and altered pericyte-endothelial interactions (O’Brown et al., 2023). Remarkably, injection of recombinant human SPOCK1 protein rescued BBB leakage in mutants, suggesting conservation of SPOCK1 function from zebrafish to humans.

Finally, glial coverage of cerebral vasculature has been observed during zebrafish brain development (Gall et al., 2025). Thus, we next focus on the cellular contributors to BBB integrity in zebrafish.

### 6.2 Astrocytes in zebrafish

Radial glial cells (RGCs), the precursors of astrocytes in mammals, were previously classified in place of astrocytes in zebrafish (Jurisch-Yaksi et al., 2020). However, Chen et al. (2020) identified *bona fide* astrocytes in zebrafish that are highly similar to mammalian astrocytes. The authors demonstrated that astrocytes derived from RGCs in the larval brain and spinal cord could be visualized *in vivo* for the first time using advanced imaging systems (Chen et al., 2020). They further reported that the transparency of zebrafish larvae allowed tracking of single RGCs and astrocytes from birth through late larval stages. Moreover, their study revealed that FGFR3/4 are required for vertebrate astrocyte morphogenesis and that astrocytes become highly branched to establish distinct territories, as shown using a cell-specific CRISPR/Cas9 system.

In mammals, the water channel AQP4 is localized to astrocytic endfeet that contact the vasculature (Corbo et al., 2012; Ikeshima-Kataoka et al., 2013). By contrast, in zebrafish, AQP4 is distributed throughout the radial glial process and rarely contacts blood vessels (Grupp et al., 2010). The differences in AQP4 expression patterns between zebrafish and mammals are thought to result from species-specific adaptations to their respective environments during evolution. Thus, *in vivo* imaging analysis using transgenic zebrafish provides unique opportunities to elucidate the details of AQP4 localization in astrocytes.

Tripartite synapses, in which astrocytes are closely associated with pre- and postsynaptic elements, have been extensively studied in rodents, where astrocytes monitor synaptic activity and actively regulate synaptic transmission (Chung et al., 2015; del Río-Hortega Bereciartu, 2020). Recently, Koh et al. (2025) identified tripartite synapses in zebrafish using GCaMP6s imaging of dorsal root ganglion neurons, spinal neurons, and astrocytes. They showed that astrocytic processes directly contacted the synaptic cleft region in the spinal sensorimotor circuit, as confirmed by electron microscopy reconstruction. These findings indicate that astrocyte-neuron interactions at the synaptic level are evolutionarily conserved across vertebrates.

The glymphatic system differs from the lymphatic system, although its function is analogous to that of the lymphatic system, was first clearly reported using *in vivo* two-photon imaging in the mouse brain (Iliff et al., 2012). In this system, cerebrospinal fluid enters the parenchyma along perivascular spaces formed by astrocytic endfeet surrounding penetrating arteries, and interstitial fluid is cleared along perivenous drainage pathways. Interestingly, a similar system has also been identified in zebrafish. Li et al. (2025) demonstrated that radial glial astrocytes mediate brain lymphatic development in zebrafish, a process modulated by neural activity. Their findings highlighted the importance of the brain’s immune system via specific glial subpopulations in zebrafish. Thus, the glymphatic system appears to be conserved at least across vertebrates.

### 6.3 Pericytes in zebrafish

Pericytes display region-specific specialization within the brain. For example, cerebral pericytes are neural crest-derived, similar to mammalians, whereas pericytes in the hindbrain vasculature originate from the mesoderm (Bahrami and Childs, 2018). Thus, the developmental origins of pericytes in zebrafish differ across brain regions. Wang et al. (2014) demonstrated that Notch3 signaling establishes brain vascular integrity by regulating brain pericyte number using zebrafish embryos and larvae.

More recently, a pericyte-targeted knock-in zebrafish line was generated using a PDGFR*β* promoter-driven P2A-Gal4-VP16 construct via CRISPR/Cas9 genome editing (Zi et al., 2025). Crossing this line with the transgenic 4x nrUAS: GFP zebrafish allows *in vivo* imaging of brain pericytes during development.

### 6.4 Microglia in zebrafish

To investigate the functional role of microglial cells in the developing brain, Wnt/β-catenin signaling was genetically reduced and compared between developing mouse and zebrafish brains (Van Steenwinckel et al., 2019). The authors demonstrated that the Wnt pathway regulates microglial activation and is critical in human brain injury. Thus, modulation of microglial activity may provide therapeutic strategies for developmental brain disorders.

Transgenic zebrafish lines carrying fluorescent reporters driven by the macrophage-expressed gene 1 (mpeg1) promoter revealed that microglia are more motile and phagocytic in larvae than in mature stages, as observed by real-time imaging (Svahn et al., 2013). Moreover, developing microglia frequently contact brain capillaries, and in the adult optic tectum (OT), microglia exhibit extensive branching, similar to mammalian microglia.

*In vivo* imaging of microglial cells in zebrafish embryos has been clearly demonstrated using apolipoprotein E locus–driven GFP (Apo-E-GFP) transgenic lines (Peri and Nüsslein-Volhard, 2008). Interestingly, the authors showed that microglia phagocytose injected Gram-negative *E. coli* bacteria in the brain and apoptotic neurons. Notably, they further demonstrated that the *α*1 subunit of the large vacuolar v-ATPase complex is required for the phagocytic and degradative activity of microglia in zebrafish. Because the authors observed “indigestion” of microglia in gene knockout zebrafish, investigating the functional consequences of microglial defects may provide critical insights into the development of degenerative diseases in humans.

A comprehensive single-cell transcriptomics analysis was recently reported for microglia, oligodendrocyte-lineage cells, and quiescent radial astrocytes (qRA) isolated from the zebrafish midbrain OT with or without stab wound injury at different developmental stages (Qin et al., 2025). The authors identified two centrally located qRA subtypes: qRA1, predominant at larval stages, and qRA2, enriched at juvenile and adult stages. Interestingly, TBI-induced qRA remained inactive following early larval TBI but showed proliferative responses at later stages. For microglial subtypes, larval-dominant MG1 cells mediated phagocytic clearance, whereas adult-enriched MG2 cells orchestrated synaptic refinement, as determined by gene ontology analysis. In addition, following TBI, three distinct reactive microglial states were identified: iMG-1, resembling activated macrophages; iMG-2, resembling regulatory macrophages; and iMG-3, corresponding to wound-healing macrophages. Unlike mammals, zebrafish possess the capacity for neuronal regeneration following TBI. Detailed investigation of these zebrafish microglial subtypes may pave the way for developing mammalian therapeutics for CNS degeneration.

### 6.5 Zebrafish models for neurodegenerative diseases

Zebrafish represent an excellent animal model for investigating drug discovery, therapeutic delivery, and treatment strategies for brain injury and neurodegenerative diseases.

In the following sections, we focus on recent findings regarding the involvement of glial cells in neurodegenerative diseases in zebrafish.

Moreover, we summarized several disease models using zebrafish and their results in Table 4.

**Table 4:** Neurodegenerative disease models of zebrafish.

Diseases	Methods	Target cells	Results	Target molecules	References
AD	A microinjection via cerebroventricular route in adult zebrafish	Whole brain	AD-like symptoms		Javed et al. (2019)
	Microinjection of casein-coated gold nanoparticles (βCas AuNPs) in AD model zebrafish	Pericyte, astrocyte	Alleviated AD-related pathology	SMAD family member 3 (SMAD3), Vascular Endothelial Growth Factor (VEGF)	İş et al. (2024)
	Excessive fat intake in zebrafish	Whole body	Cognitive impairment	Fat	Meguro et al. (2019)
	Chalcone derivative SG06 in a scopolamine-induced AD-like zebrafish model (network pharmacology and molecular docking)	Whole brain	Improved cognitive function; reduced anxiety-like behavior; restored social interactions	AKT serine/threonine kinase 1 (AKT1)	Dharshan et al. (2025)
	Neochlorogenic acid (natural polyphenolic compound) in AlCl₃-induced AD zebrafish	Neuron	Suppressed antioxidant-related proteins and Nrf2 activation improved BBB resilience	NF-E2-related factor 2 (Nrf2)	Gao et al. (2025)
	Treatment of Aβ-injected zebrafish larvae with pan-PKC inhibitor G983	Whole brain	Restored oligodendrocyte and myelin alterations to control levels	Protein kinase C (PKC)	Balantzategi et al. (2025)
	Berberine chloride (BC) treatment in AlCl₃-induced AD zebrafish larvae	Whole brain	Neuroprotective; docking results indicated BC may act as an ABCA1 agonist	ATP-binding cassette subfamily A member 1(ABCA1)	Uvarajan et al. (2025)
	Expression of human TDP-43 targeted to nucleus, cytoplasm or ALS-linked variant (G294V)	Motor neuron	Impaired motor function, motor neuron loss, and muscle atrophy	TAR DNA-binding protein 43 (TDP-43)	Hogan et al. (2025)
PD	Berberine application in zebrafish PD model	PD neuron	Neuroprotective activity	Berberine	Wang et al. (2021)
	Exposure to fluorescently labeled berberine in zebrafish PD model	PD neuron	Neuroprotective activity	Berberine	Razali et al. (2022)
	Exposure to N-acetylcysteine (NAC), resveratrol, quercetin, selenium and CoQ10, in a rotenone-induced zebrafish PD model	PD neuron	Ameliorated motor activity and restored dopamine levels	NAC, resveratrol, quercetin, selenium, and coenzyme Q10 (CoQ10)	Rahimmi and Khademerfan (2025)
	Exposure to α-Terpineol in MPTP-induced Parkinsonism zebrafish larvae	PD neuron	Neuroprotective effects	α-Terpineol	Chinnappan et al. (2025)
	A53T α-Syn transgenic zebrafish	PD neuron	Inhibition of accelerated α-Syn degradation	ATP-citrate lyase (ACLY)	Son et al. (2025)
Epilepsy	Genetic zebrafish models for seizures/epilepsy	Neuron, muscle	Brain activity measured by calcium imaging and locomotor tail-beat recordings		Cho et al. (2020a,b)
	Ischemic–hypoxic (I/H) microenvironments induction	Neuron, vascular cell	Seizure activity and vascular malformations resembling focal cortical dysplasia type II (FCDII)	Focal cortical dysplasia type II (FCDII)	Fang et al. (2025)
	phf21ab-knockout zebrafish	Neuron	Forebrain and midbrain volume reduction; epileptiform discharges	PHD Finger Protein 21A (PHF21A)	Wang T. et al. (2025)
	Nec-1 exposure in PTZ -induced seizure model	Astrocyte	Reduced seizure severity; reversed astrocytic activation	Nec-1(Necrostatin-1), Receptor-interacting serine/threonine-protein kinase 1 (RIPK1)	Ravikumar et al. (2025)
	Neuropeptide galanin exposure in PTZ-induced seizure model	Whole brain	Decreased seizure severity but paradoxical increased seizure occurrence via receptor subtype specificity	Galanin receptor 1 a (galr1a)	Rieser et al. (2025)
	grin2Aa and/or grin2Ab gene deletion	Neuron, muscle	Increased locomotor activity enhanced GluN2B-containing NMDARs in grin2A mutants	Glutamate ionotropic receptor NMDA type subunit 2A (GRIN2A)	Abramova et al. (2025)
	*In silico* and *in vivo* zebrafish modeling of DENND2B variants	Neuron, muscle	Neurodevelopmental impairment, epilepsy, hypotonia, psychosis and catatonia	DENN (differentially expressed in normal and neoplastic cells) domain-containing protein (DENND2B)	Murthy et al. (2025)
AxD	Expression of the R239C mutation in human GFAP in transgenic zebrafish	Whole body	First stable AxD transgenic zebrafish reported; GFAP protein aggregation in larvae (hallmark of AxD); impaired lipid metabolism and oxidative stress.	Glial Fibrillary Acidic Protein (GFAP)	Bellitto et al. (2025)

#### 6.5.1 Zebrafish models of AD

In one model, Aβ was microinjected into the cerebroventricular region of adult zebrafish, which produced clinically relevant AD-like symptoms (Javed et al., 2019). Interestingly, microinjection of casein-coated gold nanoparticles (βCas AuNPs) into AD model zebrafish alleviated these pathological symptoms. To clarify the molecular changes underlying BBB alterations in AD, single-nucleus RNA sequencing was performed on vascular and astrocytic cells in AD versus control brains (İş et al., 2024). The authors identified an inverse relationship between SMAD3 upregulation in AD pericytes and vascular endothelial growth factor downregulation in AD astrocytes in Ab42-treated zebrafish lines, which was further supported by pharmacological analyses. Because these altered molecular mechanisms of BBB integrity were conserved between zebrafish models and human patients, they represent potential therapeutic targets for AD pathology.

In another study, Meguro et al. demonstrated that cognitive impairment induced by excessive fat intake could be reproduced in zebrafish (Meguro et al., 2019). As described earlier in the “LD accumulation in brain” section, inadequate lipid regulation may contribute to neurodegeneration.

#### 6.5.2 Zebrafish models of PD

Berberine, an alkaloid extracted from Berberis plants, has been applied in zebrafish PD models (Wang et al., 2021). Fluorescently labeled berberine demonstrated neuroprotective activity by alleviating PD-like behaviors induced by MPTP, a neurotoxin that induces PD-like pathology (Razali et al., 2022), and by reducing dopaminergic neuronal loss. The authors suggested that berberine exerts its protective effects partly through mitochondrial mechanisms and may represent a candidate anti-PD drug.

More recently, integrative analyses using PD neurons, organoids, zebrafish, and mouse models identified ATP-citrate lyase (ACLY), a key enzyme generating acetyl-CoA in the cytoplasm, as a risk factor for PD pathology (Son et al., 2025). The authors focused on the A53T mutation in SNCA, one of the most frequent missense mutations. Inhibition of ACLY restored autophagy and reduced pathological α-Syn levels in PD neurons and organoids. Furthermore, in A53T *α*-Syn transgenic zebrafish and mice, ACLY inhibition enhanced *α*-Syn degradation. These findings suggest that ACLY may represent a promising therapeutic target for PD.

#### 6.5.3 Zebrafish models of epilepsy

Zebrafish models for seizures and epilepsy have been developed over the past two decades and are comprehensively summarized elsewhere (Yaksi et al., 2021). For instance, the ease of maintaining zebrafish across a wide range of aquarium temperatures allows hyperthermia to be used for inducing seizure models. Furthermore, genetic models of seizures and epilepsy are well established, and fluorescence calcium imaging of brain activity, combined with video recording of locomotor tail beats, takes advantage of zebrafish transparency. Adult zebrafish can also be used for epilepsy modeling (Cho et al., 2020a,b).

Fang et al. (2025) performed molecular analyses of transcriptional profiles from 15 patients to investigate mechanisms underlying epilepsy. They revealed that vascular malformations and abnormal SMCs generate ischemic–hypoxic (I/H) microenvironments, which subsequently disrupt neuronal and astrocytic activity, leading to neuronal loss. Using zebrafish models, they demonstrated that I/H microenvironments induced seizure activity, paralleling pathological features observed in human patients.

Recently, Necrostatin-1 (Nec-1), a potential anticonvulsant, was identified using the zebrafish pentylenetetrazol (PTZ)-induced seizure model (Ravikumar et al., 2025). Nec-1 inhibits receptor-interacting protein kinase 1 (RIPK1), a signaling molecule implicated in neuroinflammation and neurodegeneration. The authors showed that Nec-1 reduced seizure severity and reversed astrocytic activation in zebrafish. Given that abnormal astrocytic and microglial dysfunction has been reported in human epilepsy patients and animal models, these findings suggest that Nec-1 exerts anticonvulsant effects through its anti-inflammatory properties.

#### 6.5.4 Zebrafish model of Alexander disease

Alexander disease (AxD) is a rare astrocytic neurodegenerative disorder classified as a leukodystrophy and caused by mutations in the GFAP gene (Brenner et al., 2001).

Recently, Bellitto et al. (2025) reported the generation of a stable transgenic zebrafish line (zAxD) carrying a human GFAP mutation associated with the severe phenotype of AxD type I patients. Transcriptomic and proteomic analyses of zAxD larvae confirmed alterations in cellular respiration and lipid metabolism, along with upregulation of oxidative stress (Bellitto et al., 2025). These alterations were newly identified and had not been reported in other animal models of AxD. Thus, this transgenic zebrafish model may contribute to clarifying the mechanisms of disease onset and to the development of therapeutic approaches for AxD.

In addition, Saito et al. (2024) revealed that microglia sense astrocytic dysfunction via P2Y12 signaling to protect against AxD pathology in AxD model mice. Therefore, microglial contributions to AxD pathogenesis may soon be elucidated in zebrafish models as well.

## 7 Comparison of brain regeneration mechanisms between zebrafish and mammals

Zebrafish are capable of regenerating their brains after injury, a capacity that distinguishes them from mammals.

Self-renewing neural stem cells have been identified in the adult zebrafish telencephalon (Adolf et al., 2006; Chapouton et al., 2007). These progenitors give rise to newborn neurons that reside in the ventricular zone. Previous studies demonstrated that inflammation is required and sufficient to induce proliferation of neural progenitors and to promote neurogenesis in the adult zebrafish brain (Kyritsis et al., 2012). In the spinal cord, Hui et al. (2015) identified key pluripotency-related factors, including upregulation of pou5f1 and sox2, during regeneration after spinal cord injury. The authors suggested that elucidating these molecular mechanisms may pave the way toward therapeutic targets for mammalian spinal cord injury. In addition, FGF signaling was shown to be a critical regulator of glial cell morphogenesis in axonal regeneration. For example, glial migration was observed 2 weeks after spinal cord injury, glial bridge formation required for axonal regeneration appeared at 3 weeks, and axonal remodeling was evident by 6 weeks (Goldshmit et al., 2012). Furthermore, Notch-1 signaling plays an important role in generating neural precursor cells that migrate to the injured region and subsequently differentiate into mature neurons (Kishimoto et al., 2012).

In contrast to mammals, zebrafish rapidly induce neurogenesis after a TBI (detailed in the “Clinical Features of TBI” section). In adult zebrafish, stab wound injury to the telencephalon results in wound closure within a few days without scar formation (Baumgart et al., 2012). Thus, the zebrafish telencephalon provides a useful system to explore the mechanisms of regeneration. Consistently, another study demonstrated that telencephalic stab wounds in zebrafish revealed constitutive neurogenic activity (Pellegrini et al., 2023). Recently, molecular mechanisms of regeneration have been clarified in zebrafish TBI models. Palsamy et al. (2023) showed that microglia are essential for regeneration in the zebrafish brain, using genetic mutant lines in which microglia were ablated after brain injury. They further revealed that phosphorylated Stat3 (signal transducer and activator of transcription 3) and *β*-catenin signaling in microglia are required for telencephalic regeneration. These findings suggest that targeting these pathways may be effective for promoting repair in the injured mammalian brain.

Chen et al. (2024) compared telencephalic regeneration in zebrafish and rodents and summarized the signaling pathways that regulate inflammation and the factors controlling regeneration in the adult zebrafish brain. They pointed out that BMP-Id3 upregulation in the SVZ promotes astrogenesis, leading to gliosis and glial scar formation. Because no reactive gliosis or scar formation is observed in the zebrafish brain after injury, modulating this signaling pathway may open new avenues for promoting regeneration in the mammalian brain.

## 8 Conclusion

In the first section, studies of astrocytic and microglial molecules have shown that these cells can serve as therapeutic targets for neurodegenerative diseases in mouse models (Figure 1 and Tables 1–3). In addition, the second section highlights the importance of lipid metabolism in glial cells, particularly in astrocytes, in the context of neurodegenerative disorders (Figure 2). In contrast, the third section underscores that relatively few studies have examined glial cells in relation to neurodegenerative diseases in zebrafish models (Table 4).

Taken together, we have introduced a range of recent studies targeting glial cells that utilize the advantages of different animal models to investigate gene functions in the brain. These studies collectively demonstrate that glial cells hold considerable promise for the development of therapeutic strategies against neurodegenerative diseases.
